# Simultaneous Chloramphenicol and Florfenicol Determination by A Validated DLLME-HPLC-UV Method in Pasteurized Milk

**Published:** 2016

**Authors:** Rouhollah Karami-Osboo, Ramin Miri, Katayoun Javidnia, Farzad Kobarfard

**Affiliations:** a Medicinal and Natural Products Chemistry Research Center, Shiraz University of Medical Science.; b Department of Medicinal Chemistry, School of Pharmacy, Shahid Beheshti Medical University.

**Keywords:** DLLME, Chloramphenicol, Florfenicol, Milk, HPLC

## Abstract

The antibiotic residues in milk are a well-known serious problem and pose several health hazards to consumers. We have described a simple, rapid, and inexpensive DLLME-HPLC/UV technique for the extraction of chloramphenicol and florfenicol residues in milk samples. Under the optimum conditions, linearity of the method was observed over the range 0.02-0.85 µg/L with correlation coefficients > 0.999. The proposed method has been found to have a good limit of detection (signal to noise ratio = 3) for chloramphenicol (12.5 µg/Kg) and florfenicol (12.2 µg/Kg), and precision with relative standard deviation values under 15% (RSD, n = 3). Good recoveries (69.1–79.4%) were obtained for the extraction of the target analytes in milk samples. This simple and economic method has been applied for analyses of 15 real milk samples. Among all samples only one of them was contaminated to florfenicol; 62.4 µg/Kg and contamination to chloramphenicol was not detected.

## Introduction

The widespread use of antibiotics as growth promoting or therapeutic agents in food-producing animals may result in antibiotic residues in milk and dairy products. The main risk of consumption of the milk with antibiotic residues arises from the danger of increasing bacterial resistance (1) and also the appearance of allergic reactions to antibiotics (2). Chloramphenicol (CAP), a broad-spectrum antibiotic, was originally isolated from *Streptomyces venezuelae* in 1947 and is the first antibiotic that synthetically produced on a large scale (2). CAP has been widely used since the 1950s to treat food-producing animals. The toxic effects and the risk of aplastic anemia and carcinogenic properties of CAP are well-known (3). In 1990, the International Agency for Research on Cancer (IARC) considered CAP as “probably carcinogenic to humans” (group 2A). After 1994, the use of CAP has been banned in food producing animals by European Union (EU) and any residue must not be detected in milk samples (4). Due to the ban of using the CAP in food-producing animals, florfenicol (FLF), a synthetically produced fluorinated analogue of CAP, was developed for veterinary use to treat diseases in livestock (5). Same as CAP, use of FLF is not permitted for milk -producing animals from which milk is produced for human consumption (6). The FLF MRLs were established for muscle, liver and kidney of bovine but there is no MRL for FLF in milk (7). Contamination to CAP in milk has been reported before; in one study in Turkey, a high incidence rate of CAP and tetracycline was observed in 46.8% of milk samples (7). Dispersive liquid-liquid micro extraction (DLLME) as a fast extraction method was successfully used in biological fluids (8) and food analysis before (9). This method was applied for analysis of CAP in honey and milk (1, 10, 11). During the past few years, several methods (HPLC-UV, ELISA, LC-MS/MS, GC-MS) for simultaneous determination of CAP and FLF residue in various matrices have been proposed in which the analyte was extracted by liquid extraction (LLE), followed by clean-up by solid-phase extraction (SPE) or molecular imprinted solid-phase extraction (MIP) (5). 

In the present study, we developed a simple, rapid, inexpensive, and eco-friendly method (DLLME) for extraction of CAP and FLF from milk samples, which can be used as a routine extraction method in food quality control laboratories. The effect of various parameters on the recovery of antibiotics such as type and volume of DLLME extractant, pH and volume of water was investigated and the validated method was used for screening of 15 real milk samples.

## Experimental

*Chemicals*

Analytical standards (pestanal quality) of CAP and FLF were purchased from Sigma–Aldrich (Germany). The HPLC-grade acetonitrile, methanol, and all analytical grade extraction solvents were purchased from Merck (Darmstadt, Germany). Deionized water was prepared from a Milli-Q water purification system at 18.2 MΩ cm (Bedford, MA, USA). Individual 100.0 µg mL^−1^ stock standards of CAP and FLF were prepared by dissolving in acetonitrile. These solutions were stored at –20 °C (12). These solutions were further diluted to yield the appropriate working solutions. All standard solutions were sealed and stored at – 20 °C, protected from light, for no longer than 1 month.

*Instrumentation*

The chromatographic analyses were carried out using a Waters Breeze (Waters, Milford, MA, USA), HPLC system equipped with a binary HPLC pump (Waters 1525), Waters 717 plus auto-sampler and a Waters 2487 dual λ absorbance UV detector (Waters, Milford, MA, USA). The reverse phase column was a Waters Nova-pak^®^ C-18, 150 mm ×3.9 mm ID, 4 µM particle size (Waters Milford, MA, USA). The mobile phase consisted of water/ acetonitrile (75:25 v/v) at a flow rate of 1.2 mL min −1 and the volume injected was 100 µL. The column was maintained at 40 °C and the eluent was monitored between 0- 6.5 min at 224 nm for FLF and after 6.5 min, at 278 nm for CAP (13). Breeze software was used for controlling the system operation, collecting and analyzing the data.

*Dispersive liquid–liquid microextraction procedure (DLLME)*

A 5.00 mL portion of milk sample was deproteinized by addition of 10 mL acetonitrile and shaking for 5 min, followed by centrifugation at 9000 rpm for 5 min. A 0.4 mL chloroform as extraction solvent was add to 1.0 millilitre of the deproteinized milk (as a disperser solvent); then quickly injected into the 1 mL deionized water via a 1.0 mL Hamilton syringe (Hamilton, Reno, NV, USA). A cloudy solution (water, acetonitrile, and chloroform) was formed in the test tube. The mixture was then centrifuged for 5 min at 4500 rpm, causing the dispersed fine droplets of the extraction phase to settle to the bottom of the conical test tube. The settled extraction phase was collected using a 1.0 mL Hamilton syringe and this organic phase was dried under nitrogen stream at 30 °C. The residue was resolved in 500 μL mobile phase and 100 μL of this sample was injected to HPLC.

## Results and Discussion

*Optimization of DLLME*

In order to optimize the experimental conditions for determination of FLF and CAP by DLLME in milk, the effective parameters on extraction efficiency such as type and volume of the high density extraction solvent, salt addition and amount of water were studied by spiking the blank milk sample with 300 µg of each CAP and FLF per Kg of milk.

*Type and volume of the extraction solvent*

**Figure 1:**
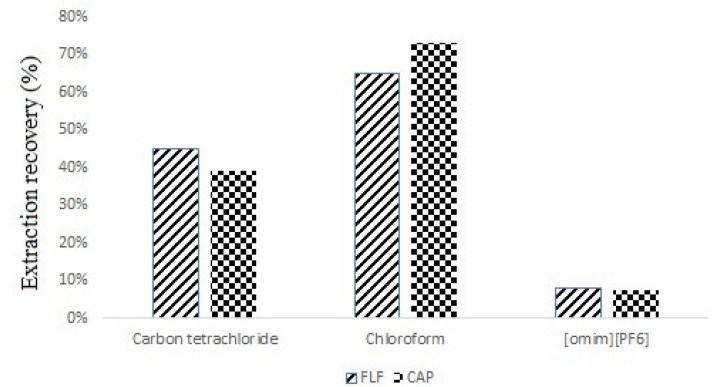
Effect of different extractant solvents on extraction recovery of spiked sample at 300 µg of each CAP and FLF per Kg of milk. Extraction conditions: water volume: 2.0 mL; Extractant volume: 0.2 mL; disperser solvent volume, 1.0 mL

Selection of high density extraction solvent is very important in DLLME procedure (14). For this purpose, carbon tetrachloride, chloroform, dichloromethane and 1-Octyl-3-Methyl-Imidazolium Hexafluorophosphate ([omim] [PF6]) (ionic liquid) were evaluated by applying 0.2 mL of each extraction solvent to the DLLME process. The 0.2 mL of each extraction solvents was added to different vials that contain 1.0 mL of deproteinized spiked milk and then, the mixture was quickly dispersed in 2.0 mL deionized water at separate tubes. No sediment phase was observed when dichloromethane was used as extraction solvent, which was due to its higher solubility in aqueous solution. The best recovery was obtained by using chloroform as extraction solvent (Figure 1.). Thereby, chloroform was selected as extraction solvent for further work.

**Figure 2:**
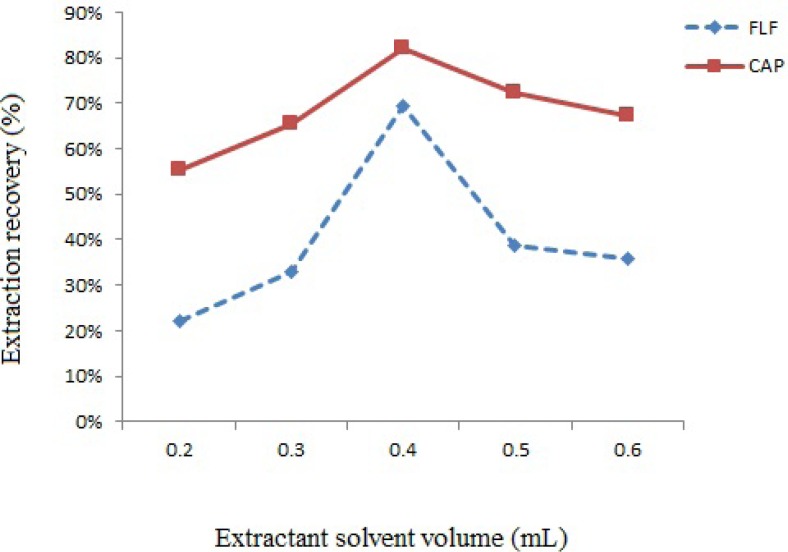
Effect of chloroform volume on extraction recovery of spiked sample at 300 µg of each CAP and FLF per Kg of milk. Extraction conditions: water volume, 2.0 mL; disperser solvent volume, 1.0 mL

In order to evaluate the effect of extraction solvent volume, different volumes of chloroform (0.2-0.6 mL) were added to separate vials that containing 1.0 mL of deproteinized spiked milk and the efficacy of extraction was evaluated (Figure 2.). By 0.4 mL of chloroform the efficiency of extraction was better than the other volumes; above 0.4 mL of chloroform, the recovery decreases probably due to decrease in the number of droplets available for extraction. Therefore 0.4 mL of chloroform was selected for subsequent experiments.

*Effect of water amount*

**Figure 3:**
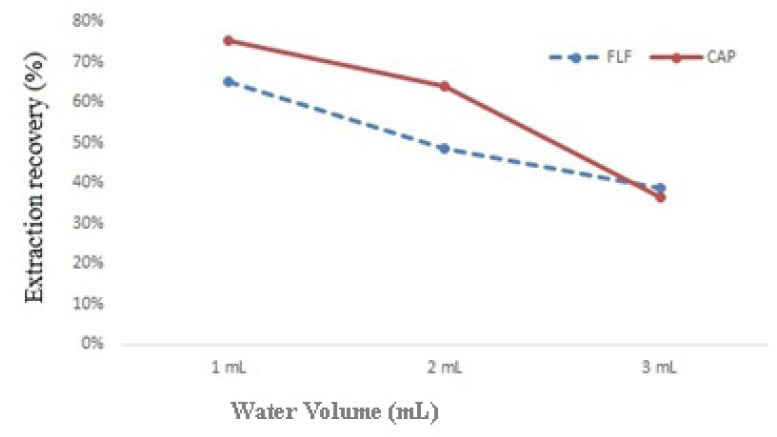
Effect of water amount on extraction recovery of spiked sample at 300 µg of each CAP and FLF per Kg of milk. Extraction conditions: Extractant volume, 0.4 mL; disperser solvent volume, 1.0 mL

At 1.0, 2.0 and 3.0 mL of deionized water, effect of water amount on the extraction recovery was studied. By increasing the volume of water from 1.0 to 3.0 mL, extraction recovery decreased. Therefore 1.0 mL deionized water was used as aqueous part of DLLME (Figure 3.).

*Effect of salt addition*

**Figure 4:**
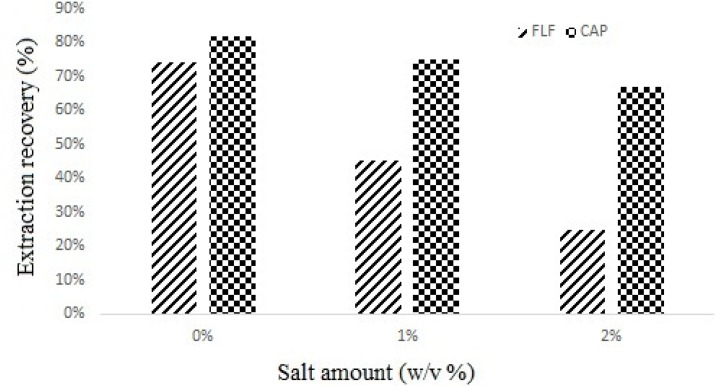
Effect of Salt addition on extraction recovery of spiked sample at 300 µg of each CAP and FLF per Kg of milk. Extraction conditions: water volume, 1.0 mL; Extractant volume, 0.4 mL; disperser solvent volume, 1.0 mL

Salt addition may have different results in DLLME like reducing (9), increasing (14) and without any effect on recovery (15). To evaluate the effect of salt addition on DLLME performance, various experiments were performed by adding different amounts of NaCl (0–2%, w/v) under constant experimental conditions, the results showed that the volume of the separated phase increased but the yield of extraction decreased (Figure 4.). Based on this result, no addition of salt was employed in all experiments.

*Effect of defatting*

The spiked milk sample was defatted by 20 min centrifugation at 9000 rpm and at -10 °C; after that the optimized DLLME procedure was applied on the supernatant. Results revealed that defatting did not have any effect on extraction recovery.

*Method validation*

Under optimized conditions, the blank milk samples were spiked at three levels, 150, 300 and 600 µg of each CAP and FLF per Kg of milk; the intra-day assay (within-day repeatability) and inter-day assay (between-day repeatability) data were obtained. The procedure was repeated in three different days and the percentage of relative standard deviation (RSDs %) were calculated. The linearity of calibration curve, limit of detection (LOD, S/N = 3)**, **limit of quantification (LOQ) and the correlation coefficient (R^2^) for CAP and FLF were studied (Table1.). In the present study, we developed a simple, rapid, inexpensive, and eco-friendly method (DLLME) for extraction of CAP and FLF from milk samples, which can be used as a routine extraction method in food quality control laboratories. The effect of various parameters on the recovery of antibiotics such as type and volume of DLLME extractant, pH and volume of water was investigated and the validated method was used for screening of 15 real milk samples.

## Experimental

*Chemicals*

Analytical standards (pestanal quality) of CAP and FLF were purchased from Sigma–Aldrich (Germany). The HPLC-grade acetonitrile, methanol, and all analytical grade extraction solvents were purchased from Merck (Darmstadt, Germany). Deionized water was prepared from a Milli-Q water purification system at 18.2 MΩ cm (Bedford, MA, USA). Individual 100.0 µg mL^−1^ stock standards of CAP and FLF were prepared by dissolving in acetonitrile. These solutions were stored at –20 °C (12). These solutions were further diluted to yield the appropriate working solutions. All standard solutions were sealed and stored at – 20 °C, protected from light, for no longer than 1 month.

*Instrumentation*

The chromatographic analyses were carried out using a Waters Breeze (Waters, Milford, MA, USA), HPLC system equipped with a binary HPLC pump (Waters 1525), Waters 717 plus auto-sampler and a Waters 2487 dual λ absorbance UV detector (Waters, Milford, MA, USA). The reverse phase column was a Waters Nova-pak^®^ C-18, 150 mm ×3.9 mm ID, 4 µM particle size (Waters Milford, MA, USA). The mobile phase consisted of water/ acetonitrile (75:25 v/v) at a flow rate of 1.2 mL min −1 and the volume injected was 100 µL. The column was maintained at 40 °C and the eluent was monitored between 0- 6.5 min at 224 nm for FLF and after 6.5 min, at 278 nm for CAP (13). Breeze software was used for controlling the system operation, collecting and analyzing the data.

*Dispersive liquid–liquid microextraction procedure (DLLME)*

A 5.00 mL portion of milk sample was deproteinized by addition of 10 mL acetonitrile and shaking for 5 min, followed by centrifugation at 9000 rpm for 5 min. A 0.4 mL chloroform as extraction solvent was add to 1.0 millilitre of the deproteinized milk (as a disperser solvent); then quickly injected into the 1 mL deionized water via a 1.0 mL Hamilton syringe (Hamilton, Reno, NV, USA). A cloudy solution (water, acetonitrile, and chloroform) was formed in the test tube. The mixture was then centrifuged for 5 min at 4500 rpm, causing the dispersed fine droplets of the extraction phase to settle to the bottom of the conical test tube. The settled extraction phase was collected using a 1.0 mL Hamilton syringe and this organic phase was dried under nitrogen stream at 30 °C. The residue was resolved in 500 μL mobile phase and 100 μL of this sample was injected to HPLC.

**Table 1:** Calibration data, LODs, LOQs and R2 of DLLME for FLF and CAP in milk

	**linear range (µg/L )**	**R²**	**LOD** **(µg/kg)**	**LOQ** **(µg/kg )**	**Spiking levels** **(µg/kg )**	**(Intra-day (n=3)**	**Inter-day (n=3)**
						**Mean recovery ± RSD (%)**	**Mean recovery ± RSD (%)**
FLF	20-850	0.999	12.2	36.6	150	78.3 ± 8.4	71.8 ± 5.5
					300	69.1 ± 6.2	72.4 ± 4.6
					600	73.7 ± 5.9	78.2 ± 3.3
CAP	20-850	0.999	12.5	37.5	150	70.7 ± 11.3	79.4 ± 8.3
					300	75.0 ± 2.4	77.2 ± 5.4
					600	72.0 ± 3.1	75.8 ± 2.1

## Results and Discussion

*Optimization of DLLME*

In order to optimize the experimental conditions for determination of FLF and CAP by DLLME in milk, the effective parameters on extraction efficiency such as type and volume of the high density extraction solvent, salt addition and amount of water were studied by spiking the blank milk sample with 300 µg of each CAP and FLF per Kg of milk.

*Type and volume of the extraction solvent*

Selection of high density extraction solvent is very important in DLLME procedure (14). For this purpose, carbon tetrachloride, chloroform, dichloromethane and 1-Octyl-3-Methyl-Imidazolium Hexafluorophosphate ([omim] [PF6]) (ionic liquid) were evaluated by applying 0.2 mL of each extraction solvent to the DLLME process. The 0.2 mL of each extraction solvents was added to different vials that contain 1.0 mL of deproteinized spiked milk and then, the mixture was quickly dispersed in 2.0 mL deionized water at separate tubes. No sediment phase was observed when dichloromethane was used as extraction solvent, which was due to its higher solubility in aqueous solution. The best recovery was obtained by using chloroform as extraction solvent (Figure 1.). Thereby, chloroform was selected as extraction solvent for further work.

In order to evaluate the effect of extraction solvent volume, different volumes of chloroform (0.2-0.6 mL) were added to separate vials that containing 1.0 mL of deproteinized spiked milk and the efficacy of extraction was evaluated (Figure 2.). By 0.4 mL of chloroform the efficiency of extraction was better than the other volumes; above 0.4 mL of chloroform, the recovery decreases probably due to decrease in the number of droplets available for extraction. Therefore 0.4 mL of chloroform was selected for subsequent experiments.

*Effect of water amount*

At 1.0, 2.0 and 3.0 mL of deionized water, effect of water amount on the extraction recovery was studied. By increasing the volume of water from 1.0 to 3.0 mL, extraction recovery decreased. Therefore 1.0 mL deionized water was used as aqueous part of DLLME (Figure 3.).

*Effect of salt addition*

Salt addition may have different results in DLLME like reducing (9), increasing (14) and without any effect on recovery (15). To evaluate the effect of salt addition on DLLME performance, various experiments were performed by adding different amounts of NaCl (0–2%, w/v) under constant experimental conditions, the results showed that the volume of the separated phase increased but the yield of extraction decreased (Figure 4.). Based on this result, no addition of salt was employed in all experiments.

*Effect of defatting*

The spiked milk sample was defatted by 20 min centrifugation at 9000 rpm and at -10 °C; after that the optimized DLLME procedure was applied on the supernatant. Results revealed that defatting did not have any effect on extraction recovery.

*Method validation*

Under optimized conditions, the blank milk samples were spiked at three levels, 150, 300 and 600 µg of each CAP and FLF per Kg of milk; the intra-day assay (within-day repeatability) and inter-day assay (between-day repeatability) data were obtained. The procedure was repeated in three different days and the percentage of relative standard deviation (RSDs %) were calculated. The linearity of calibration curve, limit of detection (LOD, S/N = 3)**, **limit of quantification (LOQ) and the correlation coefficient (R^2^) for CAP and FLF were studied (Table1.).

*Analysis of cow milk samples*

Screening of CAP and FLF for 15 milk samples, obtained from retail stores of Tehran in February 2014, was conducted by the validated DLLME method. Among all samples only one of them was contaminated with FLF (62.4 µg/Kg) and contamination with CAP was not detected.

## Conclusions

In both human and veterinary medicine, microbial resistance has been recognized as a global public health problem. The presence of antibiotic residues in milk is a serious problem and poses several health hazards to consumers. Recent studies showed that some bacteria with human origin had resistance to antimicrobial agents (8). From US$100 millions to US$10 billions per year has been the cost of direct hospital managing of antibiotic resistance in the United States (16). Use of CAP and FLF are not permitted for milk-producing animals from which milk is produced for human consumption Several extraction and clean-up methods such as LLE (17), SPE (18), MIP (19), immunoaffinity columns (IAC) (20), were proposed for extraction of CAP and FLF in milk samples, but most of them are expensive and time consuming. DLLME by ionic liquid was used before and good extraction recovery was achieved by [Bmim] BF4 (1), but here, we found that [omim][PF6] was not a good extractant and the best recoveries was obtained by using chloroform as extraction solvent. 

We have described a simple, rapid, and inexpensive DLLME technique for the extraction of FLF and CAP residues in milk samples. The LOD of method by UV detector was higher than maximum performance residue limit (MPRL) (0.3 µg/Kg for CAP) and was comparable with other researcher’s data (21), but the achievement of this research was finding a fast and valid extraction method; compared to other techniques, DLLME provides high extraction recovery within a short time with a good recovery and repeatability.
